# Book Reviews

**Published:** 1811-04

**Authors:** 


### CRITICAL ANALYSIS
OF
### RECENT PUBLICATIONS
IN THE
### DIFFERENT BRANCHES OF PHYSIC, SURGERY, AND MEDICAL PHILOSOPHY,

---

### *Stock's Life of Beddoes.*

(Concluded from p. 264.)

In 1799, Dr. Beddoes published "Contributions to Medical and Physical Knowledge, collected principally from the West of England." This volume contained Mr. Davy's original and valuable essays on light and heat. This work was speedily followed by an "Essay on the Causes, early Signs, and Prevention of Consumption, for the use of families." We forbear accompanying Dr. Stock in his copious analysis of this popular and well known treatise; and also refer those of our readers, who are interested in the effects of gaseous fluids in different diseases, to the volume before us, a considerable portion of which is devoted to that subject.

About this time Dr. B. met with another able assistant to his pursuit in Mr. King, a gentleman who to an accurate acquaintance with anatomy,

"United an insatiable ardour for physiological researches, a hand habituated to the arts of design, and a clearness of comprehension and promptness of mechanical ingenuity, which enabled him to devise a mode of executing the experiments which the doctor suggested. Nor was this a trifling task. His mind was incessantly teeming with new speculations, the accuracy of which he was eager to decide by such a test; and in cases where the means of effecting this were not at once present to his imagination, the prompt resources of Mr. King seldom failed to supply them." P. 181.

Dr. Beddoes now meditated a physiological work upon an extensive extensive scale, experiments were performed, and drawings were prepared, but the completion of the undertaking was delayed, till "the night closed upon the workman and his work."

Amongst the lighter labours of the Doctor this year, may be enumerated his proposal for subscriptions for rational toys.

"It appears, however, that it was never so extensively patronized, as to constitute an object of sufficient interest to the manufacturer, to establish a toy-shop upon this improved plan."

In the year 1801, Dr. Beddoes published a miscellaneous volume "on the Medical and domestic Management of the Consumptive, on the Powers of Digitalis, and on the Cure of Scrophula." In this volume, the author has adduced decided proofs of the advantages resulting from steadily maintaining an uniform temperature.

"The means which were made use of to secure this uniformity were various; one consisted in placing the patient in an apartment with cows. The heat and exhalation of these animals soon raised the temperature of the apartment to a genial point; at which it was steadily maintained, without much difficulty, by the occasional assistance of a stove."—P. 185.

This scheme encountered much opposition, and was generally treated with ridicule. In some instances, however, it seems to have been highly beneficial. The case of Mrs. Finch, a daughter of Dr. Priestley, is very striking. That lady, "though apparently in almost the last stage of consumption, and exhausted by sleepless nights, and almost incessant coughing, found every symptom alleviated on the second night after entering the cow-house, and enjoyed a tranquil sleep, to which she had been for some months a stranger." The second division of the volume is occupied with the effects of digitalis in consumption.

A short time after this work appeared, Dr. Beddoes, whose pen was seldom at rest, commenced a series of Essays on popular medicine. These appeared under the title of Hygëia, and were published in monthly numbers. Dr. Stock occupies nearly a hundred pages with an analysis of their contents, for which we again refer to the "Memoirs." This part of the work which we found somewhat tedious, is followed by some interesting extracts from the Doctor's correspondence: of which, however, we can only insert a very few specimens. To a young lady who, we presume, laboured under some untoward circumstances, he thus addressed himself:—

"I feel, my dear ———, what discouragement you have to thwart you; and am very far from presuming, that if I were in the same situation, ation, I could bear up under them. It all depends very much upon the power of the mind, to force itself from the present scene, and fix itself on prospects at some distance. A girl whose spirits are broken in early life, has suffered one of the greatest misfortunes. Cheerfulness and beauty set one another off; but gaiety is the charm more to be depended on for giving pleasure to those continually about you, and with regard to yourself there cannot be a question. And I believe gaiety once lost, is one of the most difficult of all things to recover. I have often witnessed the conversion of gaiety into gloom; I do not know that I ever saw the return of the former in all its original freshness and glow. How far it is possible for you to save yourself from this mental palsy, I can carcely judge, but those who fall into it can hardly be retrieved by the most prosperous circumstances. The sure way of avoiding it, is to prevent the imagination from resting on the particulars of an odiory situation. You have, I believe, as cheerful a mind as almost any of our French prisoners; you have not the prospect of a longer captivity; and you know that they have successfully relieved themselves from the horrors of their situation by the most simple of all expedients. By a knife and a bit of ivory, and often by a few straws and threads of silk, they were able to set damp, darkness, filth, short allowance, and I know not what besides, at defiance. Without doubt, had they made these nuisances the frequent subject of their thoughts and conversation, they would have been wretched in confinement, and moped or mad for life. Whether I could follow it or not, is another question; but the rule I should wish to observe in disagreeable circumstances, would be to forbid my senses to dwell upon them; and above all things, not to make them the subject of meditation, condolence, or complaint."—P. 285.

From the ease and rapidity with which Dr. B. commonly wrote, we did not expect the following confession.

"About the time I was obliged to begin the essays on health, I felt low and averse to the task I had imposed on myself. I thought I could not execute it at all. It had this striking difficulty;—that every body else had failed. I went to work *doggedly* and *dissatisfiedly*. I wrote a few lines, then took up a book, and, with a strong sense of guilt put off my labour till next day. However, by some imperceptible change, I am come to like it excessively, and look forward with delight to the hour when I am to return to it."

Yet the obligation of furnishing them by a certain time was felt by him pretty severely, as would appear from a subsequent letter, in which he says, "I am quite a slave to these Essays. The necessity of finishing each by a certain day, lies sometimes like a stone on my heart."

Doctor Joseph Frank, of Vienna, who visited this country in 1803, thus describes an interview which he had with Dr. Beddoes, at Clifton:—

"On entering his house, I gave the servant my introductory letters, that his master might be somewhat prepared, and not taken by surprize. After waiting about half a quarter of an hour, Doctor Beddoes appeared with several books under his arm. The first words that he addressed to to me were, " Which Dr. Frank are you? for there are a great many of you." Before I could answer him, he laid before me, in a row, several books, all written by Franks, constantly asking as he turned them over, " Is that you? Is that you? The first that met my eye was a Materia Medica, by Solomon Frank; I protested against this being mine. Then followed some of the works which I had written in elucidation of the Brunonian system. Having now recognized me, Brown became the first topic of our conversation. We were soon agreed upon what was worthy of praise and what of censure in that system. The conversation shortly after turned upon foreign medical literature; when I soon found that Dr. Beddoes reads German as well as he does English; and is intimately acquainted with our best authors. Among all the theories applied at present to the practice of medicine, that of Reill pleases him best. Dr. Beddoes, in his conversation, which grew every moment more interesting, shewed the same fire and animation that are observable in his writings. On this occasion, as well as at subsequent interviews, he constantly insisted upon what he had asserted in his works, with regard to the utility of various gasses and the digitalis purpurea in phthisis. He proposes to add to his former publications upon this subject.—The inspiration of the nitrous oxyd of ammonia, he says, has been beneficial in cases of paralysis. He suggested the idea of employing hydro-carbonate in strangulated hernia, with a view to throw the patient into syncope; and of attempting the reduction of the intestine, while he remained in that state. The muriate of lime is, in his opinion, superior to all other remedies in scrophulous diseases. Angina pectoris does not always exist where the coronary arteries are ossified. Puriform mucus may, in regard to its chemical composition, not essentially differ from pus. The mineral water of the hot wells near Bristol, really, now and then, cures diabetes." P. 301.

In the year 1806, after having determined upon removing to London, Dr. Beddoes was seized with severe illness. " Its more prominent features answered, with a degree of exactness not often met with in actual practice, to the nosological character of hydrops pericardii; with these, were complicated certain symptoms which marked an irregularity in the functions of the liver." The Doctor, however, was very unwilling to admit the existence of hydropic symptoms, and long combated the opinions of his attending physicians, till the complaint suddenly increased to an alarming degree, when " he immediately appeared to contemplate his situation in a desponding and almost hopeless light."

" It was now proposed to him to submit to the application of a blister of boiling water to his chest; a remedy which had been employed the year before, with signal success, in a case which he had attended with the author of this memoir, and which, although administered under circumstances almost hopeless, appeared to have been the means of preserving a most valuable life. Although he did not absolutely refuse, he begged a suspension of the proposed application till the following visit.

visit. When his medical friends next saw him, he put into their hands a written paper. He had previously been accustomed to minute down brief memoranda of his symptoms, in order to spare himself the effort of speaking, which had for some time been painful. The present paper, however, was of a different kind; it bore the evident impression of that despondence with which he reviewed his present situation. It remarked, that, in his apprehension, there existed only a very distant analogy between his case and that in which the blister of hot water had been applied*; and as he could not anticipate from it any beneficial result, he supplicated to be spared unnecessary suffering. By the time that they had perused the paper, he desired them, with an air of calmness, to witness the execution of a will, which he had hastily drawn up with his own hands. They complied with his request, and then addressed him in a style of hope and confidence; and at length, although he evidently appeared to consider their assurances as a *pious fraud*, he submitted to the application of the remedy. The effect was considerable: it was some time before the injured surface separated; and it appeared to be attended with the most beneficial consequences. A progressive amendment in his symptoms took place, which terminated in a gradual, but at length, perfect recovery. The slowness of his convalescence, however, so far abated his activity and depressed his exertions, that the immediate execution of his intended departure for the metropolis was suspended." P. 345—6.

After this he published "the Manual of Health;"—"A Treatise on Fever connected with Inflammation;"—"A Letter to Sir Joseph Banks on the prevailing Discontents, Abuses, and Imperfections in Medicine;" and a small Treatise, entitled, "Good Advice for the Husbandman in Harvest, and for all those who labour hard in hot births; as also for others who will take it in warm weather."

Towards the close of 1808, Dr. Beddoes experienced a renewal of indisposition; many of the symptoms resembled those of the former attack; and he sunk under them on the 24th of December.

The body was examined twenty-seven hours after death; but the operation was obliged to be hastily conducted, and the head was not opened. We transcribe the following particulars.

"Though Dr. Beddoes had lost a considerable portion of fat during his last illness, there remained a more than common quantity between

* This was a violent pleuritic seizure, attended with very alarming symptoms. The blister was applied in the following manner. A napkin, well heated, in order that the water might be retained as nearly as possible at the boiling point, was rolled up and forced into a pint cup, which it completely filled., leaving a convex surface rising about half an inch above the top. Boiling water was then poured on the napkin till it was thoroughly wetted. It was then hastily inverted and held for exactly thirty seconds to the patient's side. The spot where the cup was to be applied was marked, and the extent of the injury limited, by folds of linen laid on the side, covered with oiled silk, and leaving a small circular space. These precautions, however, appeared afterwards to be unnecessary.

the the skin and muscles; his stature was short, but the whole of the trunk remarkably capacious. The removal of the sternum was attended with some difficulty, in consequence of the morbid state of the pericardium, which was greatly enlarged, thickened, and to a great extent firmly connected with the costal pleura of the left side. The right lung was of an extraordinary magnitude; it was very soft, but free from any perceptible morbid changes in its structure; its surface, in several places, adhered slightly to the costal pleura, and in this side of the thorax there was a trifling quantity of coagulable lymph. The left cavity was chiefly occupied by the diseased pericardium; the left lung was almost entirely obliterated; the only remains that could be discovered of it consisted in a small, hard, and irregular substance, in which it was difficult to trace a vestige of its former organization. What remained of the costal pleura in this cavity was thickened, and a few ounces of a turbid fluid were contained in it. When the pericardium was opened, it appeared to have been nearly filled with a similar fluid, somewhat tinged with cruor. The inner surface of this membrane was not discoloured, when the coagulated lymph which adhered to it was scraped off. The heart was of a proper size, very flacid in its muscular as well as its membranous parts; no ossifications could be discovered in any part of it; but the coronary arteries near their origins seemed to be somewhat thickened. The foramen ovale was perfectly open, and the left pulmonary veins and arteries were wholly obliterated The diaphragm, except where it was connected with the pericardium, was healthy. The liver which adhered slightly to the abdominal muscles, was rather small, of a florid colour over the whole convex, and of a deep purple over the concave part of its surface; when divided in several parts no deviation from a healthy state was observed in its texture. The lobulus spigelii was very large and of a florid colour. The ductus hepaticus and communis presented one single tube of the same diameter in its whole length, and somewhat wider than usual. The gall bladder and its duct were so completely disorganized by some very early change, that the impervious remains of the latter could scarcely be traced to a small bunch of a thick and corrugated membrane in the situation of the cyst. This substance was divided, and in its centre was found a very small empty cavity of an irregular form. The stomach was very large and considerably inflamed from the cardia towards the middle; but the other portion, on the side of the pylorus, was quite healthy. The pancreas was perfect; the spleen very large and full of blood. The kidnies were both above the common size, particularly the right, both perfect in their structure and free from any morbid appearance. The rest of the abdominal viscera appeared healthy." P. 413.

We conclude this article with a spirited character of Dr. B. which appeared in the Edinburgh Medical and Surgical Journal.

" The reputation of Doctor Beddoes, as a physician, has not yet attained so high a rank as it deserves. There is an ardour of talents, an animating earnestness, a stimulant exaggeration in his writings, well adapted to arouse the torpor and to provoke the attention of medical readers. He had the mind of a poet, and a painter, and displayed the powers of his imagination in vivid representations of facts and theories. He was a pioneer in the road to discovery. He was full of enterprize, and free from prejudice, and, above all, he shewed an ardour for improvements, a disinterested sincerity, a love of truth, which shunned neither trouble nor reproach. It is to be regretted, that he suffered himself to be carried away with expectations of benefit which he thought would arise from every new hypothesis, and every untried remedy. Some of his wildest sallies were made in search of means for preventing disorder of the lungs; but although his learning has done much in making known the destructive ravages of that malady, his efforts in proposing a method of treatment has not yet been attended with the happiest success. There is a sobriety in the English people, which leads them to receive every new proposal with caution, and if it be found not to answer all that is said of it, they are apt to reject it altogether, and view future attempts with more steadiness and distrust. Dr. Beddoes possessed *satis eloquentiæ sapientiæ parum*, for the times in which he lived. Those high views, and that habitual appeal to the classical minds of philosophers which he uniformly displayed, have not obtained such sanction as they ought; his zeal has been mistaken for presumption, but perhaps some future age will affix to it the juster character of energy and truth. He was a man of great learning, and understood perfectly the Greek, Latin, French, Italian, Spanish, and German languages. His temper was admirable, and he was highly respectable in all the relations of private life."

---

*Considerations respecting the expediency of establishing an hospital for Officers on Foreign Service; suggested by the Writer's experience during the late occupation of Walcheren. By* A. B. FAULKNER, Fellow of the London College of Physicians; Physician to his Majesty's Forces; and Physician in ordinary to H. R. H. the Duke of Sussex. 8vo. pp. 16. Murray.

DR. FAULKNER was one of the physicians employed on the memorable Walcheren expedition, and consequently had ample experience of the miseries which visited the better half of our army on that pestilential shore. In the Preface to this pamphlet, he suggests the propriety of ministers communicating to the medical board, the destination of any expedition which they may deem fit to send out. The neglect of this precaution has been fatally evidenced in almost every large equipment of troops which has left this country; and, most especially so, in the late occupation of Walcheren. On this subject Dr. Faulkner might have expatiated in stronger terms. He simply remarks, that had the Medical Board known the expedition was destined to Walcheren, a greater number of medical officers would have been furnished, and various means might have been devised to mitigate gate the severity, if not to limit the extent, of the diseases which raged among our gallant troops.

The object of the present publication is to recommend the institution of a hospital for officers, when on foreign service. The advantage of such an establishment was strongly impressed on Dr. Faulkner, from the scenes of distress and fatality which he was compelled to witness during his short residence at Walcheren. The want of it was severely felt by both the patients and the medical staff in that disastrous campaign, where

> "——————————Despair
> Tended the sick, busiest from couch to couch,
> And over them triumphant death his dart
> Shook, nor delay'd to strike."

The officers were quartered in billets at such a distance from the general hospital, that they were deprived of prompt medical assistance, and care of every kind, which was necessary for their condition.

> "The time of a medical officer was wasted in long and circuitous visits to his patients; it was therefore for the most part impossible to prescribe for them in proper time; and medicines could not be prepared and brought from the general hospital when they were most necessary."

The attention of the physician was required, early in the day, at the general hospital, and it was often very late before he could see the officers for the first time: so that before their medicines could be prepared, the time for the evening visit had arrived.

During the absence of their servants for the purpose of procuring medicines from the general hospital, great inconvenience and distress were often felt from the want of proper attendance.

> "The constant and laborious attention required in cases of extreme urgency, often unfitted the servants for their duty. They were frequently under the necessity of watching night and day without relief—a task to which no constitution could be long competent; they were, therefore, sometimes taken ill, and sent to the hospital, leaving their masters altogether destitute of assistance." P. 3.

The cases which Dr. F. relates in support of his argument, are truly deplorable; and amply demonstrate the necessity of establishing the institution which he recommends.

The only objections which have been urged against it, are, '1st. That such a species of accommodation would not accord with the feelings and habits of British officers: 2dly. That the establishment would be too expensive.'

For the first of these there is no foundation; Officers of every rank in the service, have unanimously expressed their approbation approbation of a plan, which they must regard as highly conducive to their comfort, and their safety. Many of them, indeed, have offered to subscribe from their own purses towards its formation. To object to it on account of expense, is neither politic, nor consistent with the British character. That, which, it is proved, will conduce to the convenience and the health of our officers, can hardly be deemed an unnecessary expence, and ultimately must be economical, by more speedily restoring them to the service. A similar institution has long existed in the French army; and an opportunity for trying the experiments at home, occurred on the return of Sir John Moore's army from Spain, when the sick officers were accommodated in a ward of Haslar hospital. On that occasion, they "expressed an unqualified approbation of the treatment and accommodation of every kind which they received."

Dr. Faulkner has offered the sketch of a plan for a hospital, in which the officers may have all the convenience of a separate apartment, without the indelicacy of being exposed to the view of each other; and without an interruption to the free *circulation of the air*, by means of *screens*, which are intended partly to surround every bed.

The only objection which occurs to us, is that in cases where the sufferer is severely wounded, complete repose, and seclusion from all noise and disturbance must often be requisite: but how can this be attained in a large open ward, where, although the beds may be concealed by screens, the groans and exclamations of the sufferers must pervade the whole extent of the chamber?

---

### *Ramsden on the Diseases of the Testicle.*

(Continued from P. 277.)

It is generally understood, and we yet believe with truth, that the fluid found in the *tunica vaginalis testis*, when in a healthy quantity, is deposited there for the purpose of lubrication. Mr. Ramsden thinks that the exhalents have another, and more important office: and though he admits that lubrication of the cavity does thus take place, he contends, with hypothetical refinement, that these vessels serve as a waste pipe to the accidental or occasional distentions of the testicle.

"An important office of the exhalent vessels of the tunica albuginea appears to me to consist in the readiness of relief which they afford, through the means of effusion, to the testicle, whenever it is distended, or disturbed, either from natural causes or from disease. I farther consider the cavity of the tunica vaginalis to be peculiarly constructed as a receptacle receptacle* into which the excretory vessels may instantly empty themselves at all times when such sudden or immediate relief is required; so that notwithstanding the absorbent vessels may be provided with powers to meet such emergencies, yet the effusion being in many instances sudden and more rapid than the absorption, an undue proportion of fluid will, for a short period at least, occasionally remain in the sacculus without being noticed.

"Without such means of self protection, it seems to me probable that an organ so delicately constructed as the testicle would, at an early period of life, be endangered by the influence of venereal passions, or destroyed by other causes of accidental excitement. And if these provisions really exist, they will not only explain many phenomena which occur in the sudden variations of the quantity of fluid within the tunica vaginalis; but as they evince the extreme sensibilities of the testicle, will afford farther proof of its liability to become deranged by such a slight and subtle source of irritation, as I have before stated to be frequently concealed within the urethra."

The first division of this second part of the volume, as we would call it, treats of

*The acute Hydrocele.*

This disease is described as consisting of a sudden effusion of watery fluid into the tunica vaginalis testis, attended with pain.

"For the most part it occurs in persons of nervous and irritable habits, generally when they are placed under circumstances of venereal excitement, and *never but in such as have stricture, or whose urethra is in some degree in an unhealthy state.*"

It may seem fastidious to doubt what the author means by "venereal excitement;" but as it is essential to have the language of science unequivocal, and perfectly perspicuous, we make the remark. The adjective venereal, has an extensive application. Venereal excitement possibly conveys an idea of a different kind to what the author intended; even the word excitement is at times so used as not to give a distinct meaning. When the latent irritation is the result of preternatural action, *or of excitement*, (p. 41) has this defect.

It is more satisfactory to select what is practically useful. The description and treatment of the complaint comes under this denomination.

---

* Other organs, which are liable to be suddenly overcharged with blood, and distressed by distension under passion or great exertion of the body, probably possess a similar provision. I do not think it unreasonable to suppose, for instance, that the cavity of the pericardium and the ventricles of the brain, even in their healthy state, are occasionally receiving effusions for the temporary relief of their respective organs, when those organs are placed under circumstances above described.

"The

"The commencement of the acute Hydrocele is marked by severe suffering in one of the testicles (I have never seen both glands affected), darting with great violence along the spermatic chord, and extending to the back and loins. The scrotum, without becoming inflamed, is soon distended to a considerable size; the pulse is quickened, and sometimes irregular; but the tongue seldom becomes dry or furred: neither is the skin heated, as in common inflammatory attacks.

"In bulk and outward appearance, and so far as the part is painful on being handled, this complaint very much resembles hernia humoralis, from which, however, it is in fact very different, and on acconnt of requiring another mode of treatment, ought to be carefully distinguished.

"In the acute hydrócele the testicle is not enlarged or diseased, but being in a very irritable state, becomes extremely painful when any pressure is made upon the already distended tunica vaginalis. In the hernia humoralis the bulk of the tumor consists of an inflammatory enlargement of the compages of the gland itself.

"Acute hydrocele, if the tumor be not too painful to bear such a test, will necessarily be characterized by elasticity and fluctuation, and generally by transparency. In hernia humoralis the tumor will be firm, possess a considerable degree of resistance, and always be impervious to light.

"I have in some instances made the experiment of pouring cold water upon the acute hydrocele, but as cold constringes the scrotum, and thereby produces upon the testicle an effect similar to pressure, such a proceeding has greatly aggravated every symptom.

"Bleeding from the arm, which might be suggested as a proper and useful measure in hernia humoralis, is in acute hydrocele unnecessary, and in the habits in which the latter most commonly occurs does mischief.

"The principle of practice should comprehend those means which are best calculated to allay general irritability, and particularly to produce local relaxation, since the pressure of the effused fluid against the irritable and tender testicle is the chief cause of the distressing pain, with which this complaint is always accompanied.

"The treatment I have found most beneficial is, to apply leeches (proportional in number to the violence of the symptoms) to the scrotum, and when they have fallen off, to direct the patient to sit in the warm hip bath for a proper time; a brisk purgative may then be given, and after its full operation, a free dose of laudanum combined with some sudorific medicine. In cases in which the pain is peculiarly distressing, I have found it necessary to give the anodyne, without waiting the effect of a cathartic, and in some few instances to direct the farther use of laudanum in an enema.

"On the patient's getting from the warm bath into his bed, the whole of the distended scrotum should be enveloped in a large warm bread and water poultice, and should be kept carefully suspended.

"By such means I have never failed within twelve hours, and frequently in less time, to remove all the painful symptoms; and when they subside the patient has only a common transparent hydrocele, which which is generally within a short though uncertain period spontaneously absorbed.

"I have before remarked that this complaint is dependant on stricture, or on some more latent unhealthy state of urethra; it will therefore always be necessary when the excitement has subsided to direct the attention to the treatment of the urethra by the bougie, as the only means of securing the patient against a recurrence of similar distressing symptoms, whenever he may accidentally be again exposed to causes of excitement.

"Acute hydrocele constitutes one of those cases dependant on an unhealthy state of the urethra, in which an early introduction of the bougie will be injudicious. A reasonable time (perhaps two or three weeks) should be allowed to afford an opportunity for the urethra to recover a more quiescent state, and for the fluid to be absorbed before the bougie be used: but in case the hydrocele has not disappeared at the expiration of such period, it may be suspected that the existing mischief in the urethra has become permanently active, and continues to excite effusion, and the bougie should, then, be resorted to as the probable means of completing the cure of the hydrocele. In those cases in which the fluid is absorbed within the above period the application of the bougie will be adviseable as a proper precaution, and the only security against a relapse."

The next section is of great importance, and describes several complex forms of disease in the testicle and tunica vaginalis, under the general term,

### *Spurious Hydrocele.*

"Morbid enlargements of the testicle are frequently accompanied with collections of watery fluid within the tunica vaginalis, and thus constitute a very extensive class of diseases. These mixed cases have been mnch too generally considered under the term hydrosarcocele, a name which solely belongs to an incurable affection of the gland; and by the indiscriminate use of which a testicle has doubtless been subjected to extirpation, when under another denomination it would have been deliberated upon, and perhaps eventually recovered. The morbid enlargement of a testicle, accompanied with watery fluid within its tunica vaginalis, may be sclerocele, varicocele, sarcocele, scirrhus, venereal, as it has been called, or scrofulous; and though some of these states may by an intelligent surgeon be recognized through the body of the fluid, or immediately upon the tunica vaginalis having been drained of its water, yet the characters of the disease of the gland are sometimes too much confounded to make it prudent for the surgeon to attempt to designate such disease until it has been subjected to certain tests of surgical treatment."

The object of this section is to shew, and of great importance it is, that many cases of enlarged testicle complicated with fluid effusion into the tunica vaginalis are curable, notwithstanding the received opinion that the diseased gland under under these circumstances should be removed; but we must allow Mr. Ramsden to speak for himself.

"When a testicle, after the evacuation of the fluid from a supposed true hydrocele, is discovered to be in any way or to any extent enlarged and hardened, whether the spermatic chord does or does not partake of the induration, instead of proceeding to the excision of the part, as has been hitherto too frequently done, it would be proper to have recourse to the bougie for the purpose of ascertaining how far the affection of the gland may be dependant upon an unhealthy state of the urethra; and if any unhealthy state of the urethra be discovered, it is incumbent upon the surgeon to wait the result of the judicious treatment of that membrane before he pronounces the disease of the testicle to be incurable.

"The introduction of the bougie should be also resorted to in those cases of spurious hydrocele in which the enlargement of the testicle can be recognised by examination through the fluid in the tunica vaginalis, even before the evacuation of the fluid. From my own experience I can state, that when this practice has been adopted, the necessity of evacuating the water has in many instances been superseded, and under the simple treatment of the urethra by the bougie the testicle has not only been restored to its healthy state, but the undue accumulation of water has been removed by the natural powers from the tunica vaginalis.*

Six cases annexed to this section explain the author's opinions, and illustrate his practice.

The last divisson of this work treats of the

*Chronic or true Hydrocele of the Tunica Vaginalis Testis.*

In this section, which is more elaborate than either of the preceding, the author inquires into the causes of the true hydrocele, and examines the operations employed for its cure. The same idea that excited states of the urethra, are a frequent cause of disease in the testicle and its tunic, is extended to the chronic hydrocele.

"As I have in the preceding pages endeavoured to demonstrate that the acute and the spurious hydrocele are dependant on excitement within the urethra, I shall now offer a few observations in support of an opinion that the chronic or true hydrocele is produced by a similar cause under further modifications.

"We have already seen that the testicle will become hardened and enlarged, and that the sacculus of the tunica vaginalis will be distended with watery fluid in consequence of various degrees of excitement within the urethra, from the irritable and acutely painful stricture, down to that concealed, subtle, and local derangement of the mem-

* In some very few instances the fluid has remained after the reduction of the enlarged gland by the treatment of the urethra; but when this has been the case the simple tapping of the tunica vaginalis has, so far as my experience goes, always proved sufficient to the radical cure of such remaining hydrocele.

brane brane which is totally free from pain, until it is pressed upon by the bougie, and which may exist for years without the patient being conscious of its presence.

"It cannot therefore be unreasonable to suppose, that an habitual susceptibility of the whole membrane of the urethra may, in some instances, be induced by general or local causes, and although it create no conscious sensation to the patient, may have the power of gently provoking the excretory vessels of the testicle somewhat beyond their natural action, and thus by destroying the balance of fluid, in course of time establish that undue accumulation which characterizes the chronic or true hydrocele.

"I am induced to offer this opinion from a variety of facts which have presented themselves to my observation, and which lead me to suspect that in almost every case of true hydrocele, the urethra will be found either to have been exposed at some previous time to excitement or inflammation, or to be in a present state of increased sensation, from constitutional irritability, from the membranous fence, or from some other of the several general or local causes to which I have in the preceding pages referred the derangement of this membrane.

"The prevalence of hydrocele in the East and West Indies, instead of being attributable to the relaxation of the climate, may more reasonably be referred to the constant excitement to which the urethra is exposed from the habits of the table; since it is well known that in those hot countries every individual indulges in high seasoned dishes, and in the most stimulating description of diet.

"The frequency of the hydrocele being present with varicocele may also be satisfactorily explained, by referring to that state of continual excitement which is kept up by the distension and weight of the loaded vessels. In the more advanced states of varicocele nothing is more common than an habitual gleet or weeping from the urethra, which is occasioned by the dragging of the varicose vessels; and it certainly appears not difficult to suppose that such excitement in the urethra, when once established, may in its turn re-act upon the testicle, and produce a case of hydro-varicocele."

Though Mr. Ramsden is strongly impressed with his favourite hypothesis, he candidly acknowledges, that "in cases which present themselves under all the appearances of true hydrocele, in which the urethra is less obviously deranged, that an attempt to cure the complaint by the use of the bougie, will lead to no other result than an unnecessary waste of time."

The radical cure of hydrocele has been attempted, and indeed, often effected by various means; as by cautery, incision, excision, caustic, tent, cannula, seton. discutients, and injection. All these Mr. Ramsden rejects, except the last, upon which he says,

"I consider what is termed the radical cure by injection, the only method which ought to be practised in this disease: because it is not attended by those severities and risks which attach to all other modes, and is generally so satisfactory in its final result. Its superiority is indeed in every point of view so conspicuous, that I have long ranked it as one of the most perfect surgical operations we are in the habit of performing."

Though a decided preference is given to the treatment of hydrocele by *injection*, the principle upon which it has been adopted is rejected. The obliteration of the cavity of the *tunica vaginalis testis*, which has been considered as a *sine qua non* in the radical cure, our author contends is neither essential to that cure, nor does it always or frequently happen, unless the curative process has been carried to unnecessary severity.

"The effect of throwing injection into the tunica vaginalis is of two kinds. If a very strong stimulating injection be used and retained within the cavity for an unreasonable length of time; it occasions a painful enlargement of the testicle, and places the patient under all the distressing symptoms of the hernia humoralis.

"If, on the other hand, an injection be used of more moderate strength, be retained but a short space of time, and special care be taken to prevent the cannula of the trocar from rubbing against the testicle; it produces a slight inflammatory excitement of the excretory vessels without any enlargement of the gland itself, and places the patient under symptoms very much resembling those I have stated to be present in acute hydrocele.

"In the former instance, where the inflammation is so severe as to occasion the hernia humoralis, the surface of the gland and the surface of the tunica vaginalis are in contact with each other, and frequently become adherent.

"In the latter instance, where the injection produces an effect nearly similar to acute hydrocele (which is the only effect a judicious operator should aim at), and the surface of the gland and the surface of the sacculus are carried away from each other by the immediate effusion, and any adhesion between them is mechanically impossible."

The fact of the surface of the gland and the surface of the sacculus being kept apart by an intermediate effusion, is considered to be ascertained by a transparency of the tumour existing during the whole process of cure.

For the support of the opinion that the cure of hydrocele, when effected by discutients, does not arise from the application exciting an increased action of the absorbents, but from a suppression of the "excretory vessels," we must refer to the work itself; as well as for a detail of the process of cure by injection:. and be content with observing that the principle of this cure rests on exciting a moderate degree of inflammatory action.

The cases of aneurysm, with which the volume concludes, are, a case of axillary aneurysm, in which the subclavian artery was tied behind the clavicle. Case of aneurysm of the femoral artery, for which the artery was tied immediately below low Poupart's ligament. Case of popliteal aneurysm. Case of aneurysm of the femoral artery between the ham and the centre of the thigh.

---

Art. I. *Observations on two distinct Varieties of Ophthalmia, prevalent in the Army.* By Henry Walker, M. D. Assistant Surgeon, 2d Batt. 71st Regt. *The Edinburgh Medical and Surgical Journal. No.* 25, 8*vo. Edin.* 1811.

The disease of which Dr. Walker here treats, prevailed to a great extent in the 71st Regiment. From the 22d of April to the 24th of June, 114 persons were admitted into the hospital with it, out of 600 men and boys of which the battalion consisted.

This malady is stated to have attacked in two forms or varieties, modified by circumstances, which are not pointed out. An attempt to distinguish these varieties by the terms acute and chronic, he asserts, would only mislead. The first variety is thus described.

" In the place where the redness is originally discovered, the disease first gains its height, and the vessels of the tunica conjunctiva, by their minuteness and number, give to the eye something of the appearance called *chemosis*, (chymosis,) though that state has not yet taken place; for, at this time, on pressing the eyeball with the finger, the eyelid being interposed, and removing it quickly, the whole surface becomes white, and, for the first time, the vessels of the sclerotic coat are seen increased in size, carrying red blood, and running in straight lines towards the cornea. On removing the presure, these are first filled, and present a purple rather than red tint, immediately afterwards; those of the conjunctiva more tortuous and large, are longer of filling, and become of a deep red. Soon after the first appearance of the symptoms now detailed, purulency commences, constituting, according to those who have described this disease, its second stage, at which time the eye puts on an appearance which I have not seen described, nor have I ever remarked it in the more violent forms of inflammation, to which the organ is liable from external injury. In different points, under the conjunctiva, covering the eyeball, small patches of a scarlet hue appear of different sizes and irregular figures, which no pressure can remove, and which, I apprehend, are formed by extravasated blood; these, by a very gradual increase, unite, and thus chemosis is formed. This state of the eye gives to the countenance of one affected with it a very striking appearance, bnt I am far from wishing it to be understood, that this account of the early appearance of chemosis, is drawn out with the desire of its forming a pathognomonic feature; certainly, the most prominent symptom is the very early and copious effusion of a purulent-like fluid, though this can with difficulty be admitted as characteristic of the complaint, since it is by no means uncommon in the more severe attacks of inflammation of the eye, from great violence done to that organ, as in the operation of couching and extracting."

In this variety of ophthalmia, it has been observed that exposure of the eye to light, is not productive of much uneasiness. This Dr. Walker considers to be a fact of high importance as affording ground for a diagnosis: but if he intends to explain how it happens that the eye in this disease bears the stimulus of light, his description is deficient in perspecuity.

In every case of this variety of ophthalmia, it was observed that,

"The sclerotic coat had an increased vascularity, a fact which has been overlooked from the symptoms having been drawn out at an advanced period of the disease."

Copious bleeding, repeated as often as the pain required, was attended with the happiest effects. In every case where this treatment was adopted, the patients escaped derangement of vision.

"The second variety of attack commences in the whole surface of the convex conjunctiva; in the course of a very few hours, that membrane becomes loaded with enlarged vessels, running tortuously towards the cornea, which in a little time may be seen affected, to the extent of a line in its whole circumference, with a slight elevated vascular border; the eyelids, of a red colour in the place occupied by the conjunctiva, feel stiff and uneasy; exposure of the affected organ to light gives considerable irritation; but it seldom happens that pain warns the patient of his complaint; lachrymation, but no purulent discharge takes place; and chemosis is never present, nor can the vessels of the deeper seated membranes be discovered affected, as in the former variety. If the disease be permitted to go on, and, as I have noticed, still more if venesection be employed, it presents day after day the same appearance; the eyelids become thickened, and slightly everted; and the cornea begins to suffer, either from the encroachment of the border affecting its circumference, already described, or from the deposition of matter between its laminæ, and consequent ulceration, hypopion, and staphyloma, generally pretty near to the place corresponding to the pupil.

In the first variety copious bleeding from the system afforded the surest relief; in this, so far from curing, it tended to weaken the vessels of the conjunctiva, and render the disorder more obstinate. Scarifying the vessels of the tunica conjunctiva, and dropping immediately afterwards on the eye-ball a stimulating material, is recommended. Dissappointed in every method, our author endeavoured to make the eye, affected with this variety, assume the inflammatory action of purulent ophthalmia, and then applied the usual remedy venesection. This practice is said to have proved successful, not a single enlarged vessel remaining at the end of a fortnight.

Art. 2. *An Essay on Staphyloma pellucidum conicum.* By Robert Lyall, Surgeon, Paisley.

Mr. Lyall considers the first notice of this disease to have occurred in Leveille's French translation of Scarpa's work on Diseases of the Eye. It has since been slightly noticed by Dr. Edmonstone, and more distinctly by Mr. Wardrop, who describes the disease at some length in Essays on the morbid Anatomy of the human Eye.

The *Staphyloma pellucidum conicum*, is here considered as being nothing more than an alteration in the form and size of the cornea, in consequence of an increased quantity of aqueous humour being contained in the anterior chamber of the eye; and the same causes are assigned for this increase of fluid, as those which give rise to dropsies in general. This complaint is characterized by the conical form of the cornea, its peculiar lucidness, and the derangement of vision. It is considered as incurable by Mr. Lyall; but attempts were made to relieve it, by puncturing the cornea, and by the application of astringent collyria. The confused state of vision may be partially corrected by the employment of concave glasses; the power of those employed has been from No. 4, to 20,16. Four cases of the disease are given.

---

Art. 3. *Case of Tetanus successfully treated.* By Mr. Grimstone, Surgeon, Royal Navy.

This case of Tetanus arose from a pistol-ball passing through the palm of the hand, and fracturing the middle metacarpal bone. The cure is attributed to the combined effects of ardent spirit and opium.

---

Art. 4. *Brief Outline of a Plan for diminishing the Prevalence and fatal Tendency of Hooping-cough.* By H. Edmonstone, Surgeon.

Mr. Edmonstone forms his plan for diminishing the prevalence and severity of Hooping-cough upon certain peculiarities of action to which the contagion of that disease is known to be subjected.

" One of the best established and most remarkable of these peculiarities in the history of hooping-cough, is, that the severity and danger attending the disease, are in the inverse ratio of the age of the patient; or, in other words, the younger the patient, the greater is the hazard to which health and life are exposed. In proportion as the constitution acquires vigour and stability, the danger becomes comparatively trifling; and after the age of puberty ceases almost entirely, together with the susceptibility susceptibility of the disease. Another circumstance, with regard to the operation of the contagious principle, particularly deserving of attention, is, that hooping-cough is observed to be much milder in warm than in cold climates; and it would seem to be in conformity to this law, that the disease is found to be more severe in this country during autumn and winter, than during spring and summer. It is not necessary here to enter into any discussion respecting the causes of these differences, in the degrees of danger and severity, arising from age, climate, or season. It is sufficient that experience has established the fact of their uniform occurrence. The two important practical rules which result immediately from them, and to which I am, on the present occasion, particularly anxious to direct the attention of the public, are the following:

I.—*To use every precaution to guard against the hooping-cough, during the very early stages of life.*

II.—*To avoid its attack during the unfavourable season of the year.*

To ensure a selection of the favourable period with respect to age and atmospheric temperature, it is proposed that every one having the disease shall wear such a distinguishing mark as will enable those who are liable to take it, to keep beyond the sphere of contagion.

---

Art. 5. *Inquiry into the Exactness of some Statements made by Messrs. Nagle and Wilson, Naval Surgeons, respecting their extraordinary Success in the Treatment of the Yellow Fever.* By a Naval Surgeon.

In the second volume of Dr. Currie's Medical Reports, Mr. Nagle gives an account of the employment of cold affusion in a fever that occurred on board the Ganges, in the West-Indies. One hundred and twenty cases of this disease, denominated Yellow Fever by Mr. Nagle, and of which, under this treatment, only two persons died. Mr. Wilson, Surgeon of the Hussar, gives (Trotter's Medicina Nautica) a relation of a still more successful employment of cold affusion in a fever, also called Yellow Fever, on board that vessel: for of eighty-three cases none were fatal.

The object of this naval surgeon's inquiry is to point out the probability of these gentlemen having mistaken the disease.

*(To be continued.)*

